# Aquaporin 4 and brain-related disorders: Insights into its apoptosis roles

**DOI:** 10.17179/excli2021-3735

**Published:** 2021-06-01

**Authors:** Ehsan Dadgostar, Vida Tajiknia, Negar Shamsaki, Mojtaba Naderi-Taheri, Michael Aschner, Hamed Mirzaei, Omid Reza Tamtaji

**Affiliations:** 1 Department of Psychiatry, School of Medicine, Isfahan University of Medical Sciences, Isfahan, Iran; 2 Student Research Committee, Isfahan University of Medical Sciences, Isfahan, Iran; 3 Department of Surgery, School of Medicine, Iran University of Medical Sciences, Tehran, Iran; 4 Psychiatry and Behavioral Sciences Research Center, Mashhad University of Medical Sciences, Mashhad, Iran; 5 Students' Scientific Research Center, Tehran University of Medical Sciences, Tehran, Iran; 6 Department of Molecular Pharmacology, Albert Einstein College of Medicine, Bronx, NY 10461, USA; 7 Research Center for Biochemistry and Nutrition in Metabolic Diseases, Institute for Basic Sciences, Kashan University of Medical Sciences, Kashan, Iran

**Keywords:** Aquaporin 4, apoptosis, brain diseases

## Abstract

Brain-related disorders are leading global health problems. Various internal and external factors are involved in the progression of brain-related disorders. Inflammatory pathways, oxidative stresses, apoptosis, and deregulations of various channels are critical players in brain-related disorder pathogenesis. Among these players, aquaporins (AQP) have critical roles in various physiological and pathological conditions. AQPs are water channel molecules that permit water to cross the hydrophobic lipid bilayers of cellular membranes. AQP4 is one of the important members of AQP family. AQPs are involved in controlling apoptosis pathways in brain-related disorders. In this regard, several reports have evaluated the pathological effects of AQP4 by targeting the apoptosis-related processes in brain-related disorders. Here, for the first time, we highlight the impact of AQP4 on apoptosis-related processes in brain-related disorders.

## Introduction

Brain-related disorders represent significant health problems (Olesen et al., 2012[53]). The multifactorial pathophysiology of brain-related disorders has yet to be fully understood. Shedding further light on their mechanisms may improve treatments, affording new targets for pharmacological interventions. Several clinical risk factors have been recognized, and multiple cellular and molecular pathways and targets have been hypothesized as triggers of brain related disorders (Hughes et al., 2012[28]), such as inflammation, apoptosis, and aquaporins (AQPs), to name a few (Kouchaki et al., 2017[39], 2018[38]; Shah et al., 2003[70]; Tamtaji et al., 2019[82]). 

The apoptotic pathways have essential roles in the pathophysiology of brain-related disorders. Apoptosis is an orderly autonomous death process controlled by several genes (Peter, 2011[56]), aimed at removing dying cells and avoid tissue damage (Ravichandran, 2011[62]). In recent years, apoptosis has been evaluated as a therapeutic target for different neurological disorders such as Alzheimer's disease, stroke (Alam, 2003[1]; Sheng et al., 2009[72]), and brain tumors, such as gliomas (Iwamaru et al., 2007[32]). Various cellular and molecular mechanisms are associated with apoptosis in brain-related disorders, with channels and cell transmitters (e.g., aquaporin 4) playing significant roles in regulating apoptosis (Jablonski et al., 2007[33]; Tamtaji et al., 2019[82]). 

Aquaporins are water channel proteins (Preston et al., 1992[59]). Structurally, AQPs are composed of eight membrane-embedded domains, of which six are membrane-spanning and their N- and C-termini are in the cytoplasm (Ho et al., 2009[24]). AQP4 are involved in the pathophysiology of brain-related disorders (Lee et al., 2012[42]; Xu et al., 2015[94]), as for example, in spontaneous recurrent seizure in AQP4-deficient mice (Lee et al., 2012[42]). Further, AQP4 deficiency has been shown to exacerbate cognitive disorders and Aβ aggregation in an animal model of AD (Xu et al., 2015[94]), as well as other brain-related disorders (Chu et al., 2014[14]; Ding et al., 2013[17]). Chu et al. (2014[14]) reported that AQP4 deletion increased the rate of apoptosis after intracerebral hemorrhage in mice. This effect was mediated by increased cytokine expression, such as TNF-α and IL-1β, which initiated the apoptotic cascade and activated caspase-3 and -8. Another study has shown that IL-6 mediated the increase in apoptosis induced by interferon-α, likely by the down-regulation of AQP4 in human hippocampal progenitor cell line HPC0A07/03C (Borsini et al., 2017[6]).

Several studies have evaluated the relationship between AQP4 and the pathophysiology of brain-related disorders (Lee et al., 2012[42]; Xu et al., 2015[94]). Given the reported role of AQP4 in controlling apoptotic pathways (Zheng et al., 2017[101]; Chu et al., 2014[14]), this novel review addresses current knowledge on the relationship between AQP4 and apoptosis in brain-related disorders.

## Mechanisms Associated with Apoptosis

**Figure 1:**
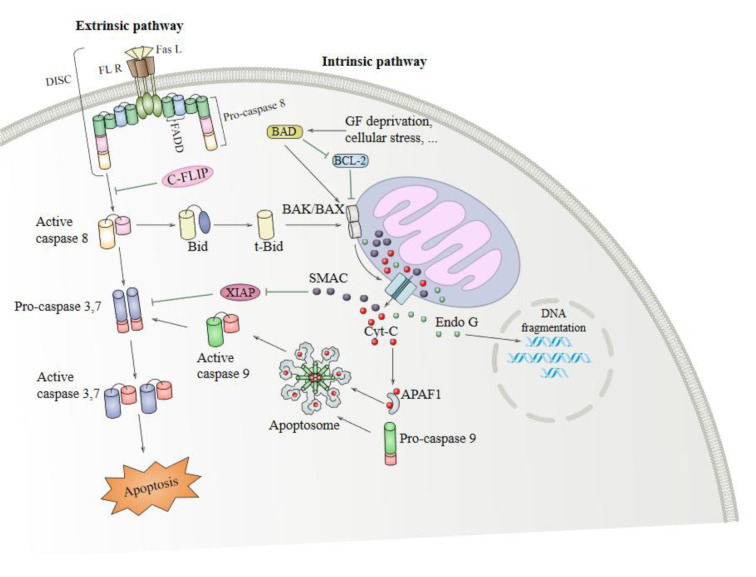
Apoptosis-associated cellular and molecular pathways

There are two main apoptotic signaling pathways (the extrinsic and intrinsic pathways), mutually affecting each other (Figure 1(Fig. 1)) (Igney and Krammer, 2002[30]). The extrinsic pathway begins in the cell surface where various ligands and death-related receptors are located (Puviani et al., 2003[61]; Lan et al., 2011[41]; Wang et al., 2008[90]). Tumor necrosis factor α (TNF-α) is a member of the TNF superfamily of ligands; it is a pro-inflammatory cytokine that has an important role in the pathophysiology of several diseases (Victor and Gottlieb, 2002[86]). The extrinsic signaling pathway begins with transmembrane receptor-mediated interactions through the TNF receptor gene superfamily; it has a cysteine-rich extracellular domain and a cytoplasmic domain composed of 80 amino acids. The cytoplasmic or death domain transmit signals related to cell death from the cell surface to the intracellular signaling pathways (Ashkenazi, 2002[4]; Wang and El-Deiry, 2003[91]; Wiens and Glenney, 2011[92]). Interaction of TNFR1-associated death domain protein (TRADD), a 34 kDa protein with TNFR1, occurs following TNF binding to TNFR1 (Hsu et al., 1995[26]). In addition, FAS (CD95) and FASL ligand (CD95L), as well as CD40 and membrane-bound CD40L, have essential roles in the extrinsic apoptosis signaling pathway (Walczak and Krammer, 2000[88]; Rudi et al., 1998[63]).

The intrinsic signaling pathway initiates apoptosis via non-receptor-mediated stimulation that is activating intracellular signals that influence targets within the cell, particularly in the mitochondria. The intrinsic pathway is regulated by B-cell lymphoma protein 2 (Bcl2) family proteins causing the release of cytochrome c from mitochondria leading to activation of caspase-9 through cytosolic apoptosome complex formation with apoptotic protease activating factor-1 (Apaf-1) (Shakeri et al., 2017[71]). In turn, caspase-9 leads to activation of caspase-3/6/7 and DNA fragmentation (Slee et al., 2001[75]; Porter and Janicke, 1999[58]). In addition, different proteins, including Bcl2 family proteins like Bax/Bak, induce mitochondrial membrane permeabilization, leading to the release of pro-apoptotic proteins from the mitochondria (Ruffolo et al., 2000[64]; Newmeyer and Ferguson-Miller, 2003[49]; Schuler and Green, 2001[68]; Gross, 2016[22]). These pro-apoptotic proteins such as apoptosis inducing factor (AIF), Caspase-Activated DNAse (CAD) and (Smac)/DIABLO and HtrA2/Omi leads to DNA fragmentation (Hong et al., 2004[25]; Joza et al., 2001[35]; Li et al., 2001[44]; Fulda, 2015[21]). 

## AQP4

**Figure 2:**
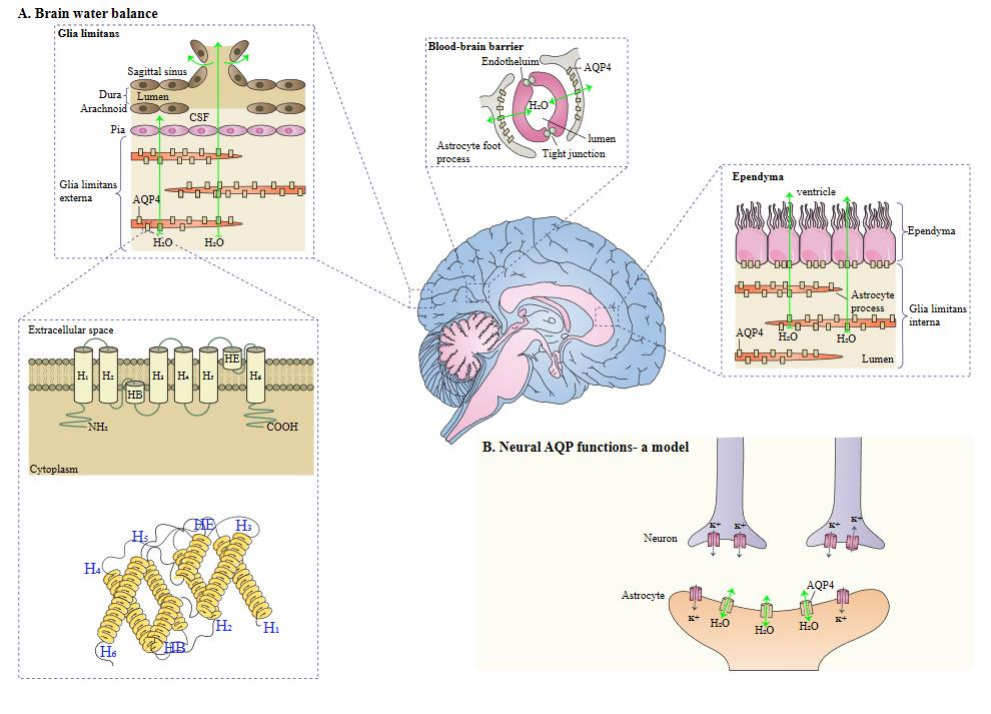
A schema of the role of AQPs. A. AQP monomers composed of helical domains surrounding a narrow aqueous pore. Monomers assemble to form tetramers in the membrane. B. AQP4 facilitates water movement into and out of the brain across brain-fluid barriers at locations indicated. This figure was adapted from Verkman (2011).

In humans, two main isoforms of AQP4, including M1 (known as a long isoform) and M23 (known as short isoform) exist. The two isoforms are generated by alternative splicing. M1 translation begins at Met-1, and for M23, translation begins at Met-23 (Yang et al., 1995[95]; Lu et al., 1996[46]), Orthogonal arrays of particles (OAPs) are supramolecular crystalline assemblies formed by the aggregation of AQP4 tetramers (Verkman et al., 2013[84]). AQP4 protein is located in glial cells, ventricles, blood vessels, subfornical organ, and supraoptic nucleus (Nielsen et al., 1997[51]; Satoh et al., 2007[66]). In astrocytes, AQP4 is expressed in the end-feet surrounding blood vessels (Hubbard et al., 2015[27]). In addition, AQP4 is colocalized with the proteoglycan brevican, which is expressed in cerebellar astrocytes (Hubbard et al., 2015[27]). Notably, when water enters via the lamellipodium into the cytoplasm driven by an osmotic gradient, astrocyte migration can be enhanced by AQP4. AQP4 has also a crucial function in the neuroexcitation, secondary to neuronal release of isoosmolar K+, followed by its uptake and water by astrocytes on the other side of the synaptic cleft (Papadopoulos and Verkman, 2013[54]). AQP4 has different functional roles, such as regulation of body water balance and water flow and the K+ reuptake. Astrocytes in AQP4−/− mice show enhanced tracer coupling, leading to improvement in the re-distribution of [K+]o in the hippocampus (Strohschein et al., 2011[79]). Deletion of astrocytic connexins triggers down-regulation of AQP4 expression and a reduction of perivascular AQP4 (Katoozi et al., 2020[36]). The expression profiles of AQP4 have confirmed this protein facilitates the movement of water between brain and blood and also between cerebrospinal fluid (CSF) and the brain parenchyma (Solenov et al., 2004[77]). AQP4 is vital for the maintenance of blood-brain barrier integrity (Zhou et al., 2008[103]), and it accelerates neuronal activity and astrocyte migration in brain (Tait et al., 2008[81]). High-mobility group box 1 indirectly up-regulates AQP4 expression microglia-astrocyte interaction (Ohnishi et al., 2014[52]). Figure 2(Fig. 2) (Reference in Figure 2: Verkman, 2011[85]) illustrates structure and function of AQPs. 

## AQP4 Deficiency and Apoptosis in the Brain-Related Disorders

### Alzheimer's disease

Alzheimer's disease (AD) is a leading cause of dementia (Prince et al., 2013[60]). Extracellular accumulation of amyloid β (Aβ) peptides and hyper-phosphorylation of tau proteins play an important role in the pathogenesis of AD (Grundke-Iqbal et al., 1986[23]; Dronse et al., 2017[18]). *In vitro* studies have shown that Aβ induces neuronal apoptosis (Estus et al., 1997[20]; Josepha et al., 2001[34]; Kienlen-Campard et al., 2002[37]), and that the c-Jun N-terminal kinases (JNK)-Fas ligand-Fas pathway mediates Aβ-induced apoptosis (Morishima et al., 2001[48]). Aβ significantly decreases the expression of Bcl and elevates Smac release and activation of JNK (Yao et al., 2005[99]). In addition to reducing the expression of Bcl-2, Aβ increases Bax expression (Paradis et al., 1996[55]).

AQP4 mediates synaptic plasticity and spatial memory processes (Scharfman and Binder, 2013[67]). AQP4 expression is linked to astrocytic pathology and amyloid deposition in transgenic murine models of AD (Yang et al., 2017[96]). Furthermore, AQP4 redistribution facilitates the formation of reactive glial and astrocyte structural plasticity and decreases neuropathology in mouse models of AD (Smith et al., 2019[76]). AQP4 deficiency exacerbates memory deficits and brain oxidative stress (Liu et al., 2012[45]), and polymorphisms in AQP4 are predictive of amyloid burden and disease stage progression. For example, the *AQP4* SNP rs151244 is associated with increased Aβ deposition (Chandra et al., 2021[9]).

**Figure 3:**
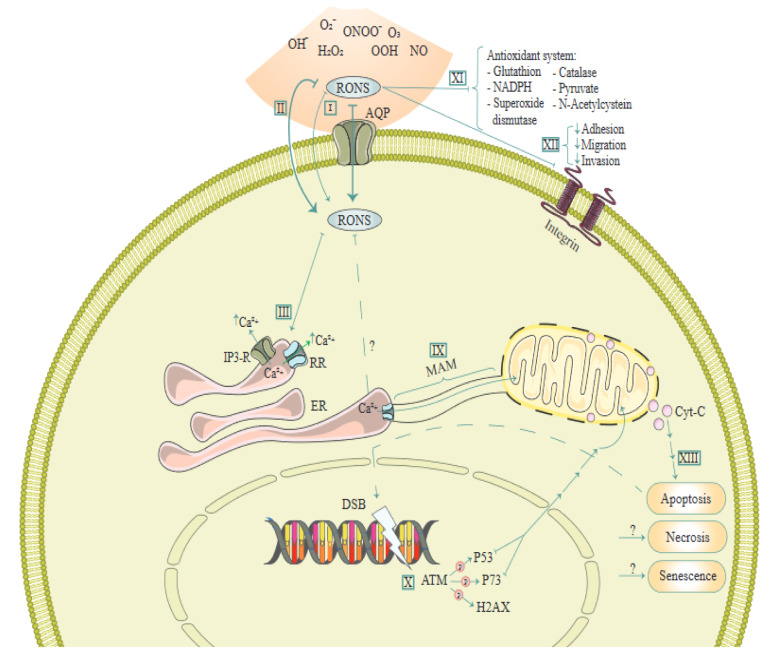
A schema of molecular mechanisms involved in the effect of reactive oxygen and nitrogen species (RONS) via Aquaporins (AQ). This figure is adapted from Semmler et al. (2020).

AQP4 knockdown has been shown to decrease Aβ(1-42)-induced apoptosis and activation in cultured astrocytes, which was related to a diminished uptake of Aβ secondary to decreased up-regulation of low-density lipoprotein receptor-related protein-1 (Yang et al., 2012[98]). In human hippocampal progenitor cell line, HPC0A07/03C, IL-6 has been shown to mediate increased apoptosis induced by interferon-α, potentially via down-regulation of AQP4 (Borsini et al., 2017[6]). Figure 3(Fig. 3) (Reference in Figure 3: Semmler et al., 2020[69]) illustrates molecular mechanisms involved in the effect of reactive oxygen and nitrogen species (RONS) via AQs. 

### Ischemia

Cerebral ischemic disease, a common form of stroke, remains one of the leading causes of morbidity and mortality worldwide (Virani et al., 2020[87]). Ischemia increases caspase-3 expression and neuronal apoptosis (Deng et al., 2019[15]). Bcl-xL has a neuroprotective effect against brain ischemia (Cao et al., 2002[7]). Elevation of AIF, cytochrome c and Smac/DIABLO is also inherent to cerebral ischemia (Kratimenos et al., 2017[40]).

In mice, AQP4 deficiency manifests with astrocytic dysfunction after focal cerebral ischemia (Shi et al., 2012[73]). AQP4 has been implicated in cerebral water transport and the formation or resorption of edema fluid from the brain parenchyma (Bloch and Manley, 2007[5]). Manley et al. (2000[47]) have shown that AQP4-deficient mice had better neurological outcome and attenuated brain edema after focal ischemic stroke. AQP4 deficiency exacerbated ischemia/reperfusion injury with an increase in infarct size, loss of CA1 neurons, and hypertrophy of astrocytes in mice (Zeng et al., 2012[100]). In addition AQP4-deficient mice displayed greater neuronal loss and microglial activation, concomitant with increased neutrophil infiltration in the brain after focal cerebral ischemia (Shi et al., 2012[74]). Pretreatment with TGN-020, an AQP4 inhibitor, markedly reduced brain edema and the size of cortical infarction in a model of brain ischemia (Igarashi et al., 2011[29]).

Several studies have demonstrated that AQP4 expression directly correlates with apoptosis activation. Zheng et al. (2017[101]) reported that overexpression of miR-145 inhibited apoptosis via AQP4 in a cerebral ischemic stroke model. Another study reported that AQP4 deficiency significantly reduced cell apoptosis and increased astrocytic health in cerebral ischemic stroke (Zheng et al., 2017[102]). Pirici et al. (2018[57]) reported lower number of caspase-3-positive cells following administration of TGN-020, an inhibitor of AQP4 in an animal model of ischemic stroke.

### Intracerebral hemorrhage

Intracerebral hemorrhage (ICH) is known as the most common hematoma stroke sub-type. Clinical presentation is diverse according to the location and size of hematoma and intraventricular extension of hemorrhage (An et al., 2017[2]). Apoptotic pathways have an important role in ICH, associated with caspase-3 and Bcl-2 family expression changes (Chang et al., 2014[10]; Sun et al., 2017[80]).

AQP4 SNP rs1054827 has been shown to be associated with ICH and increased perihematomaloedema volume (Appelboom et al., 2015[3]). AQP4 deletion elevates intracerebral hemorrhage damage, including neuronal death/TUNEL-positive cells, blood-brain barrier damage, and edema formation (Tang et al., 2010[83]). VEGF has been shown to decrease brain edema and neuronal death after ICH, likely related to up-regulation of AQP4 via ERK and JNK pathway activation (Chu et al., 2013[13]). Erythropoietin (EPO) has been shown to protect the blood-brain barrier integrity upon ICH concomitant with AQP4 activation secondary to p38-MAPK and JNK binding to the EPO receptor (Chu et al., 2014[12]). In addition, curcumin, a flavonoid, significantly reduced brain edema in an animal model of ICH by reducing AQP4 expression (Wang et al., 2015[89]).

In addition, a reverse correlation between AQP4 and activation of apoptotic pathway has been reported. Chu et al. (2014[14]) reported that AQP4 deletion elevated apoptosis after ICH in mice, and that the underlying mechanism was mediated by several cytokines, such as TNF-α and IL-1β, which, in turn, initiated the apoptotic cascade and activated caspase-3 and -8.

### Gliomas

Gliomas are one of the most common tumors of the CNS. Patients suffering from malignant gliomas are rarely prognosed, limiting efficient and timely treatments, and by inference, cure (Castro et al., 2003[8]). In glioma, Bcl-2 levels are increased, and apoptosis is decreased via suppression of caspase-3 and -7 signaling (Stegh et al., 2008[78]). In addition, Fas, FasL and caspases-8 are inhibited in tumor glioma cells (Saggioro et al., 2014[65]). Unlike other brain-related disorders, apoptosis induction has been advanced as a treatment strategy for glioma (Ehtesham et al., 2002[19]).

AQP-4 mRNA expression is decreased in tumor specimens of glioblastoma patients without seizures (Isoardo et al., 2012[31]). AQP4 is involved in cell migration and invasion of glioblastoma (Ding et al., 2011[16]). It has been posited that connexin 43 (Cx43) is a downstream effector for AQP4, although, AQP4 and Cx43 operate via two distinct mechanisms in triggering brain edema in the course of gliomas (Li et al., 2015[42]). The redistribution of AQP4 in glioma cells is a reaction to vascular endothelial growth factor (VEGF)-induced vasogenic edema for facilitating the reabsorption of excess fluid (Yang et al., 2012[97]). Overexpression of miRNA-320a leads to the inhibition of cell migration and invasion by targeting AQP4 (Xiong et al., 2018[93]). Glioma radiotherapy and chemotherapy have been shown to down-regulate AQP4 expression in tumor sample, contributing to resolution of brain edema (Nico et al., 2009[50]). Temozolomide (TMZ), an effective drug for glioma, has been shown to control invasion and proliferation of malignant glioma by inhibiting AQP4 expression secondary to the activation of p38 signaling in glioma cell lines (Chen et al., 2017[11]). Thus, induction of apoptosis may represent a therapeutic strategy for the treatment of glioma (Ding et al., 2013[17]). 

## Conclusions

Increasing evidence has indicated that AQP4 has a dual role in the control of apoptosis signaling in brain-related disorders. In the present review, we summarized the relationship between AQP4 and apoptosis in brain-related disorders, including AD, cerebral ischemic stroke, intracerebral hemorrhage, and glioma (Figure 1(Fig. 1)). Taken together, AQP4 is a mediator of brain pathogenesis, exerting its effects by targeting apoptosis-related processes. Future assessments on the relationship between AQP4 and apoptosis are required in hope of establishing a novel target for pharmacological approaches in the treatment of glioma and other CNS disorders.

## Notes

Hamed Mirzaei and Omid Reza Tamtaji (Students' Scientific Research Center, Tehran University of Medical Sciences, Tehran, Iran; E-mail: tamtaji.or@gmail.com) contributed equally as corresponding authors.

## Competing interests

The authors declare no conflict of interest.

## Funding

No specific source of funding is associated with this work. 

## Authors' contributions

HM, ED, MA, MN-T and O-RT contributed in the conception or design of the work and drafting of the manuscript. All authors confirmed the final version for submission.
